# Supplementary material for: Separation of Hydrochloric Acid and Oxalic Acid from Rare Earth Oxalic Acid Precipitation Mother Liquor by Electrodialysis
Source: Membranes (Basel). 2023 Jan 27;13(2):162. doi: 10.3390/membranes13020162 (PMC9964671; doi:10.3390/membranes13020162)
Supplement: Supplementary file 1 [file membranes-13-00162-s001.zip › membranes-2099303-supplementary.pdf]

membranes

Supporting information

Separation of Hydrochloric Acid and Oxalic Acid from Rare Earth Oxalic Acid Precipitation Mother Liquor by Electrodialysis

Hengcheng Zhou [1,2,3], Peihai Ju [2,3], Shaowei Hu [4], Lili Shi [1,2,3], Wenjing Yuan [2,3], Dongdong Chen [2,3], Yujie Wang [5] and Shaoyuan Shi [1,2,3,5,*]

1 College of Resources and Environment, Nanchang University, Nanchang 330031, China
2 Ganjiang Innovation Academy, Chinese Academy of Sciences, Ganzhou 341119, China
3 Jiangxi Province Key Laboratory of Cleaner Production of Rare Earths, Ganzhou 341119, China
4 Technology Center of Angang Steel Co., Ltd., Anshan Liaoning 114009, China
5 Institute of Process Engineering, Chinese Academy of Sciences, Beijing 100190, China
* Correspondence: syshi@ipe.ac.cn

1. Water absorption ratio

The water absorption ratio (parameter 7) was calculated. Cut a small piece of membrane material and immersed it in deionized water. After 72 hours, the membrane was taken out and the water on the membrane surface was completely sucked dry with filter paper. The weight at this time was weighed and denoted as m_1. Then the membrane was placed in the air-blowing drying oven at 30 °C to dry to constant weight, so as to remove all moisture in the membrane. The weight at this time was recorded as m_0. Then the water absorption ratio (W) was $W\ (\%) = \frac{100(m_1 - m_0)}{mo}$.

2 The water quality analysis report.

Table S1. The water quality analysis of the rare earth oxalic acid precipitation mother liquor.

Parameter	Unit	Quantity
COD	mg/L	28,168
TOC	mg/L	7042
Total phosphorus	mg/L	0.35
Ammonia nitrogen	mg/L	4.33
H^+	mM	1100
Ca	mg/L	6.38
Dy	mg/L	24.75
K	mg/L	282.7
Al	mg/L	242.6
Gd	mg/L	4.78
Na	mg/L	57.6
Yb	mg/L	8.7
Total iron	mg/L	30.94
Cl^-	mg/L	41650
F^-	mg/L	9.09
SO_4^{2-}	mg/L	-
NO_3^-	mg/L	468

Table S2. Hydrochloric acid purity obtained in concentrate chamber by SAEM.

Mixed Solution	Hydrochloric acid purity obtained in concentrate chamber (%)
2 M HCl + 0.4 M OA	95.7
2 M HCl + 0.6 M OA	92.6
2 M HCl + 0.8 M OA	92.0
3 M HCl + 0.4 M OA	95.8

3 M HCl + 0.6 M OA	93.2
3 M HCl + 0.8 M OA	91.8
4 M HCl + 0.4 M OA	96.7
4 M HCl + 0.6 M OA	92.5
4 M HCl + 0.8 M OA	91.5
Real oxalic precipitation mother liquid	96.0

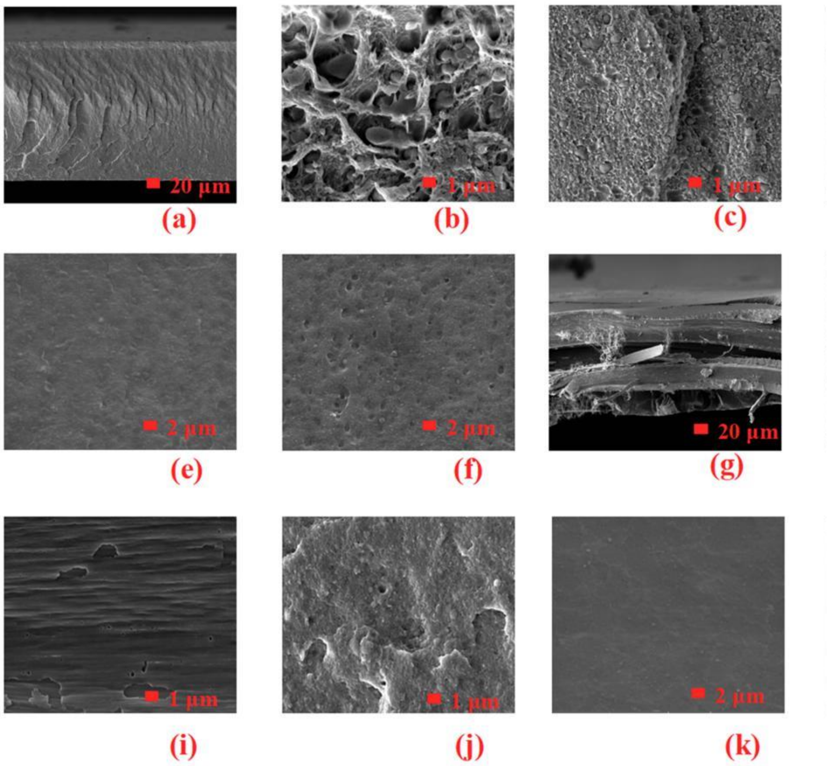

Figure S1. Scanning electron microscope (SEM) images, cross section of virgin Cation exchange membrane (CEM) (**a**)-(d), surface images of CEM used before (**e**) and after (**f**); cross section of virgin Selective anion exchange membrane (SAEM) (**g**)-(**j**), surface images of SAEM used before (**k**) and after (**l**).

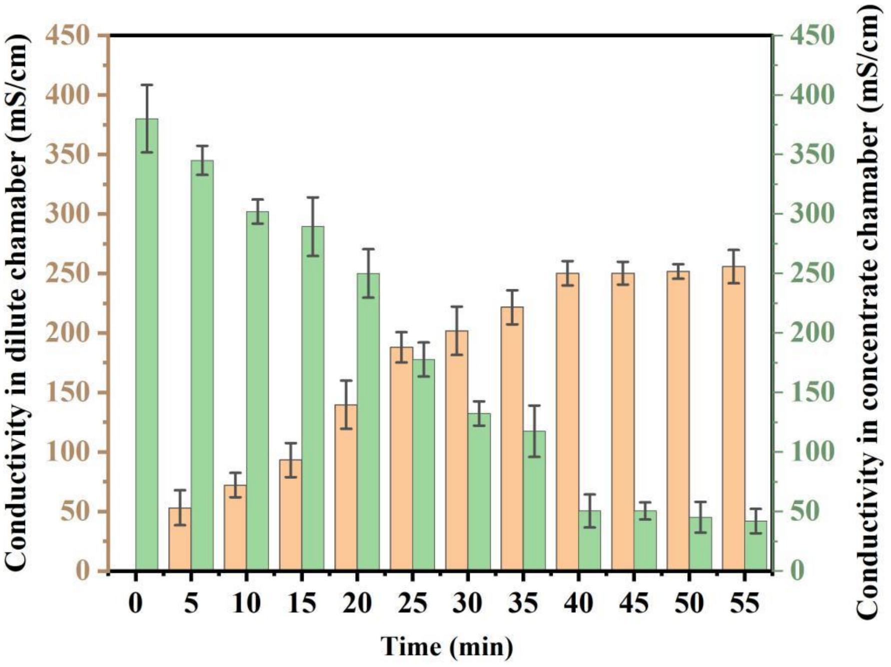

Figure S2. Conductivity of the dilute chamber and concentrate chamber.

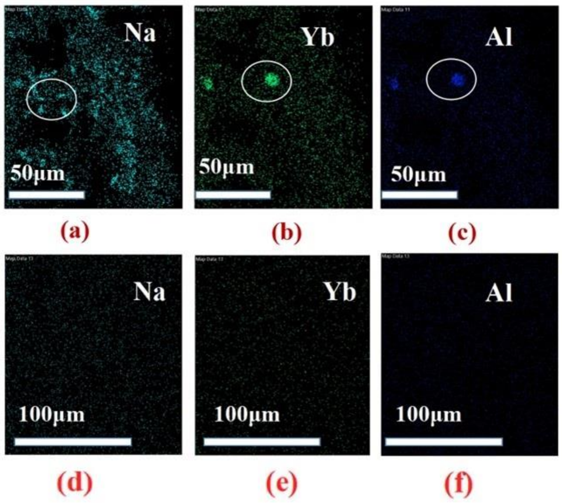

Figure S3. Fouled cross-section elements mapping of cation exchange membrane CSE (**a**-**c**) and anion exchange membrane ACS (**d**-**f**).
